# Addressing immortal time bias in precision medicine: Practical guidance and methods development

**DOI:** 10.1111/1475-6773.14376

**Published:** 2024-09-03

**Authors:** Deirdre Weymann, Emanuel Krebs, Dean A. Regier

**Affiliations:** ^1^ Cancer Control Research, BC Cancer Vancouver British Columbia Canada; ^2^ Faculty of Health Sciences Simon Fraser University Burnaby British Columbia Canada; ^3^ School of Population and Publics Health, Faculty of Medicine University of British Columbia Vancouver British Columbia Canada

**Keywords:** immortal time bias, multiple imputation, precision medicine, real‐world evidence, statistical methods

## Abstract

**Objective:**

To compare theoretical strengths and limitations of common immortal time adjustment methods, propose a new approach using multiple imputation (MI), and provide practical guidance for using MI in precision medicine evaluations centered on a real‐world case study.

**Study Setting and Design:**

Methods comparison, guidance, and real‐world case study based on previous literature. We compared landmark analysis, time‐distribution matching, time‐dependent analysis, and our proposed MI application. Guidance for MI spanned (1) selecting the imputation method; (2) specifying and applying the imputation model; and (3) conducting comparative analysis and pooling estimates. Our case study used a matched cohort design to evaluate overall survival benefits of whole‐genome and transcriptome analysis, a precision medicine technology, compared to usual care for advanced cancers, and applied both time‐distribution matching and MI. Bootstrap simulation characterized imputation sensitivity to varying data missingness and sample sizes.

**Data Sources and Analytic Sample:**

Case study used population‐based administrative data and single‐arm precision medicine program data from British Columbia, Canada for the study period 2012 to 2015.

**Principal Findings:**

While each method described can reduce immortal time bias, MI offers theoretical advantages. Compared to alternative approaches, MI minimizes information loss and better characterizes statistical uncertainty about the true length of the immortal time period, avoiding false precision. Additionally, MI explicitly considers the impacts of patient characteristics on immortal time distributions, with inclusion criteria and follow‐up period definitions that do not inadvertently risk biasing evaluations. In the real‐world case study, survival analysis results did not substantively differ across MI and time distribution matching, but standard errors based on MI were higher for all point estimates. Mean imputed immortal time was stable across simulations.

**Conclusions:**

Precision medicine evaluations must employ immortal time adjustment methods for unbiased, decision‐grade real‐world evidence generation. MI is a promising solution to the challenge of immortal time bias.

**What is known on this topic**

- Single‐arm trials evaluating precision medicine targeted therapies necessitate real‐world observational studies at risk of immortal time bias.
- While immortal time adjustment methods exist, common methods either result in information loss, involve non‐informative date assignment, or necessitate cohort definitions that risk inadvertently biasing evaluations.

**What this study adds**

- We discuss theoretical strengths and limitations of common methods for mitigating immortal time bias for precision medicine evaluations, including landmark analysis, time‐distribution matching, and time‐dependent analysis.
- We propose a new application of multiple imputation (MI) for immortal time adjustment that enhances characterization of uncertainty about the true length of the immortal time period.
- MI explicitly considers patient characteristics and their impacts on immortal time distributions, with inclusion criteria and follow‐up period definitions that do not inadvertently risk biasing evaluations.

## 1 INTRODUCTION

Regulatory and reimbursement decisions rely on evidence of safety, efficacy, clinical effectiveness, and cost‐effectiveness.^1^ Immortal time bias threatens the validity of this evidence in observational studies. Immortal time bias can occur if there is a period of follow‐up time during which intervention patients must remain outcome‐free.^2^, ^3^ For example, when patients must wait to access a genomic test after receiving a diagnosis, or when patients experience delays in accessing a targeted treatment after receiving genomic test results.^4^, ^5^ These wait times are “immortal” when a comparator group does not experience the same delay. Failing to adjust for immortal time falsely inflates time‐dependent outcomes for treated patients, including overall survival and healthcare costs.^6^, ^7^

Immortal time bias poses a significant challenge for reliable outcome measurement in precision medicine applications. For example, precision oncology studies frequently rely on single‐arm, master protocol trials for establishing safety and efficacy.^8^ By design, these trials exclude a contemporary control group for establishing comparative effects and temporal alignment of prospective data collection occurs only for intervention patients.^9^ Health system stakeholders recognize the need for real‐world data to identify counterfactuals and inform effectiveness and cost‐effectiveness for precision medicine.^10^, ^11^, ^12^ Interest in real‐world evidence signals that decisions for these technologies can be based on evidence generated in observational studies which are at high risk of immortal time bias and strongly depend on choice of cohort entry timing.^12^, ^13^, ^14^ Effective methods for immortal time bias adjustment are needed to ensure real‐world evidence is decision‐grade.

We present an overview of common methods for addressing immortal time bias, highlighting relevant strengths and limitations for precision medicine evaluations. We then propose a new adjustment method based on a novel application of multiple imputation (MI) and provide practical guidance for this approach. We illustrate the application of MI for addressing immortal time bias in a case study drawing on our previously published comparative evaluation of a Canadian single‐arm precision oncology program, and explore sensitivity to data missingness rates and sample sizes.^15^, ^16^ We conclude with a discussion of implications for future research.

### 1.1 Overview of immortal time adjustment methods

The challenge of immortal time bias is well characterized, but uptake of solutions is limited.^13^, ^17^, ^18^ In a recent review of national cancer database studies, nearly 40% of studies at risk of immortal time bias made no adjustment.^17^ These studies likely overestimated the magnitude and significance of treatment effects, providing misleading conclusions. Common solutions for immortal time adjustment include: landmark analysis; time‐distribution matching; and time‐dependent analysis.^18^, ^19^, ^20^ In the following section, we review these three approaches to immortal time adjustment and consider their applicability for evaluating precision medicine.

#### 1.1.1 Landmark analysis

Landmark analysis is common for addressing immortal time bias and is the leading approach in oncology, where precision medicine implementation is advancing.^6^, ^17^, ^21^, ^22^, ^23^ This method selects a time—the “landmark time”—at which to redefine exposure and measure follow‐up in intervention and control patients.^24^ Any patients who die or are censored before this landmark time must be excluded from subsequent outcomes analysis. While straightforward to apply, the choice of threshold is nontrivial.^25^ Landmark times are not generalizable across studies and must be chosen based on known or observed times of clinical significance.^26^ For example, the landmark time could be based on the distribution of the immortal time period experienced by intervention patients. In an evaluation of an emerging genomic test, this immortal time period may be the length of time between initial cancer diagnosis and biopsy for genomic testing. For a targeted treatment, this period could instead be time from diagnosis or relapse to treatment initiation. Any patients who die or are censored before the landmark time must be excluded from outcomes analysis, producing information loss for both intervention patients and controls. After selecting the landmark time, baseline covariate measurement, follow‐up, confounding adjustment, outcomes analysis, and results interpretation must be conducted at this new time point rather than at the observed index date.

The frequency and performance of landmark analysis in precision medicine evaluations is not systematically characterized. Research in other applications finds that treatment effect estimates are highly sensitive to the choice of landmark time.^25^ Although recommended, sensitivity analysis exploring impacts of specifying different landmark times on sample sizes, treatment effect estimates, and conclusions is uncommon.^17^, ^25^ Further, by assuming the same landmark time for all individuals in a study, landmark analysis may introduce measurement bias into outcomes analysis. In precision medicine, where genomic heterogeneity drives access to targeted interventions and treatment response, assuming a universal landmark time is inappropriate.

#### 1.1.2 Time‐distribution matching

Time‐distribution matching builds on landmark analysis by allowing variability in index dates across individuals and redefining exposure status and follow‐up periods only for control patients, reducing information loss. This approach, proposed by Zhou et al. (2005), redefines exposure status and follow‐up periods for control patients based on immortal time‐adjusted index dates observed for intervention patients.^27^ Pseudo index dates are randomly assigned to control patients based on their observed distribution among intervention patients. This method ensures that index date distributions are balanced across groups. Any controls ineligible for their assigned date are excluded from subsequent outcomes analysis. Baseline covariates must be reestimated and adjusted for in outcomes analysis based on this new index date.

In 2005, Zhou et al. demonstrated that time‐distribution matching performed comparably to other established methods addressing immortal time bias, and this approach has become common when comparing clinical and economic outcomes across groups who did not experience the same index date.^27^, ^28^, ^29^, ^30^ Yet recent simulation studies show conflicting performance for time distribution matching, with only partial mitigation of immortal time bias, particularly in the presence of low event rates.^19^ This residual bias may be partially attributable to the non‐informative nature of date assignment inherent to time‐distribution matching. This approach fails to consider intervention and control patient characteristics that may affect receipt of precision medicine when assigning index dates. As a result, endpoints and confounders may be measured inappropriately, introducing bias.

#### 1.1.3 Time‐dependent analysis

Time‐dependent analysis is an alternative that has variable uptake across clinical contexts. In oncology, a recent review found that only 5% of national cancer database studies adjusting for immortal time employed this approach, whereas in broader pharmacoepidemiology studies time‐dependent analysis is far more frequent.^17^, ^31^ Time‐dependent analysis defines and adjusts for time‐varying treatment status, for example, by using a time‐dependent Cox regression model.^17^, ^19^ Patients are classified as not‐yet‐treated while they await treatment and only factor into the treatment group after receiving treatment. Careful consideration of treatment status definitions, inclusion criteria, and patient follow‐up are, however, required for validity.^32^ Traditionally, time‐dependent analysis allows not‐yet‐treated individuals to transition between treatment groups, with their control outcomes censored at date of treatment receipt.^2^, ^6^, ^33^ Alternatively, time‐dependent analysis may allow only for delayed entry of treated patients, excluding not‐yet‐treated patients from the control group.^34^ With delayed entry, separate survival probability model estimation occurs for treated and control patients. For control patients, the model spans the entire observed follow‐up period. For eventually treated patients, the model is instead indexed to treatment initiation date. Owing to independent model estimation with delayed entry, alternative methods for obtaining standard errors for treatment effect estimates must be employed, such as nonparametric bootstrapping.^34^

Irrespective of the approach chosen for precision medicine evaluations, the bias potential introduced by modifying follow‐up periods for treated patients must be considered. For progressive diseases, such as cancer, ignoring the not‐yet‐treated period for treated patients and initiating follow‐up from a preferentially selected index date may introduce observed and unobserved time‐varying confounding because patient characteristics, such as disease severity and expected prognosis, change over time. In these scenarios, patient's probability of survival after the entry time may depend on the entry time itself, known as delayed entry bias.^35^, ^36^ Precision medicine researchers must address any introduced time‐varying confounding to ensure comparability of treated and control patients throughout the follow‐up period and achieve unbiased estimation with time‐dependent analysis.

### 1.2 Multiple imputation for immortal time adjustment

We propose MI to address limitations of existing immortal time adjustment methods. MI uses the distribution of observed data to estimate multiple values for a missing value, reflecting uncertainty around the true value.^37^ If the length of immortal time observed in intervention patients and unobserved in control patients is considered missing data, MI will predict missing values using an imputation model that considers individual patient characteristics and observed immortal time distributions. MI acknowledges and quantifies uncertainty in patient‐specific immortal time periods through imputing multiple possible index dates for control patients and reestimating treatment effects based on these dates. Using MI, we can explore sensitivity to the index date assignment mechanism and incorporate patient characteristics into immortal time adjustment. MI further avoids introducing delayed entry bias which may occur with time‐dependent analysis. MI is frequently used for imputing missing data elements in comparative evaluations, even those accounting for immortal time bias, but to our knowledge has not previously been considered for imputing immortal time.^15^, ^38^, ^39^, ^40^

### 1.3 Practical guidance

**FIGURE 1:**
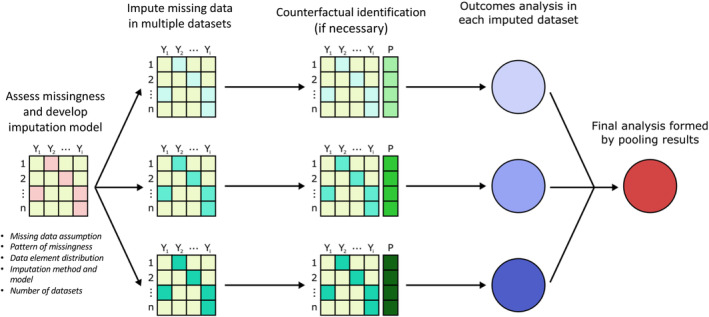
Overview multiple imputation framework. This framework depicts the sequence of steps required when multiply imputing data for comparative evaluations and highlights key considerations when developing a data imputation model.

Our proposed application of MI for inferring immortal time involves a series of steps outlined in Figure 1.^15^ When imputing missing data, steps include: (1) selecting the imputation method; (2) specifying and applying the imputation model; and (3) conducting comparative analysis and pooling estimates. Once data are imputed, counterfactual identification and subsequent outcomes analysis must occur within each dataset in which missing values have been imputed using MI (termed a multiply imputed dataset).^41^, ^42^ Estimates are then combined across datasets using a series of formulas known as Rubin's rules (Supplemental Materials).^43^ Rubin's rules require that the statistics of interest being combined adhere to normality and normalizing transformations may be necessary if this condition is unmet.^44^ Combined estimates measure variability within and across multiply imputed datasets to capture uncertainty.

#### 1.3.1 Step 1: Selecting the imputation method

To select an appropriate imputation method, missing data assumptions, the distribution of missing data elements, and relative performance of competing approaches must be considered.^38^ Data are assumed to be missing completely at random (MCAR) if neither observed nor unobserved data impact the probability that data are missing.^45^ The length of the immortal time period for precision medicine evaluations is unlikely MCAR. By design, the immortal time will be known for trial‐enrolled intervention patients, but not for comparators. Instead, the less restrictive assumption that data are missing at random (MAR) is more appropriate, where the probability of missingness may depend on observed data, such as treatment status, but not on unobserved data. MI methods assume that data are MAR and that parameters of the data model are distinct from parameters of the missing data mechanism, termed ignorability.^46^ The ignorability assumption cannot be tested given that missing data and their underlying mechanism are unobserved, but sensitivity analysis assuming different missing data models, including missing not at random (MNAR), can be examined to characterize stability of results.^47^, ^48^

In addition to considering missingness mechanisms, the appropriate imputation method depends on patterns of missing data and distributions of data elements. For example, missing data may be univariate if only the immortal time variable contains missing data, but if multiple variables are affected then the relative pattern of missing data must be considered. Missing data are monotonic if missingness in one variable determines missingness in another.^46^ Monotone missing data can be filled in iteratively using a sequence of independent univariate imputation models, such as linear or nonlinear regressions or predictive mean matching. If instead the pattern of missing data is arbitrary, then alternative methods considering all missing data elements simultaneously are more appropriate, such as MI by chained equations (MICE) or multivariate normal regression. Competing MI approaches can be selected based on their sensitivity to assumed distributions of missing data elements. For example, when imputing arbitrary missing data, specifying conditional linear and nonlinear prediction equations using MICE increases flexibility in distributional assumptions compared to a multivariate normal approach.^49^

#### 1.3.2 Step 2: Specifying and applying the imputation model

The next MI decision involves specifying the imputation model. Any covariates that will factor into the analytic model must be included in the imputation model.^38^ Inclusion of the outcome and auxiliary variables that correlate with data missingness can be considered. There is a lack of consensus on whether to include the outcome within the imputation model if a matched cohort analysis planned.^41^ Matching is designed to be a function of covariates rather than outcomes, so that repeated analyses attempting to balance covariate distributions in the matched cohort do not bias final effect estimates.^50^ Considering outcomes during imputation may deviate from this property. There is further literature debate on whether to include auxiliary variables whose correlation with missingness is less than 0.40.^51^, ^52^, ^53^ Once the imputation model is specified, the final decision prior to imputation involves the number of datasets to generate. This decision can be based on prior simulation studies examining power fall off alongside rates of missingness.^54^

#### 1.3.3 Step 3: Conducting analysis and pooling estimates

In this step, a new index date is defined based on the imputed immortal time for each patient, within each MI dataset. Baseline covariate measurement, follow‐up, covariate adjustment, and outcomes analysis are conducted at the new index date, like in time‐distribution matching or landmark analysis. Results are then combined using Rubin's rules, applying transformations for non‐normal estimates, such as complementary log‐log transformation for normalizing survival probabilities and logarithmic transformations for hazard ratios.^55^ By measuring variability within and across MI datasets, outcomes estimate based on MI analysis will reflect uncertainty in imputed immortal time periods.

### 1.4 Precision medicine case study

In our recent comparative evaluation of a Canadian single‐arm precision oncology program, we employed MI and time distribution matching for avoiding immortal time bias.^15^, ^16^ In this study, we determined the comparative effects of a single‐arm precision oncology initiative applying whole‐genome and transcriptome analysis (WGTA) in patients with advanced cancers.^15^ While research‐based genomic sequencing initiatives are often designed to benefit future patients,^56^, ^57^, ^58^ our prior evaluation sought to understand downstream impacts of research participation for enrolled patients and healthcare systems. We compared early‐stage effects of WGTA on patient management, patient survival, and health system costs with usual care.^15^, ^16^ We drew on an observational, matched cohort design and population‐based administrative data to define this usual care counterfactual.

Below, we describe the methodological approach for our previous evaluation, providing additional details on immortal time adjustment methods used and outcomes comparisons. We reproduce our original results and emphasize their implications for immortal time. We then report on a simulation study examining results sensitivity to varying both sample sizes and data missingness rates.

## 2 METHODS

### 2.1 Study design

Our retrospective, matched cohort study design was conducted between July 2014 and December 2015. While these data are >5 years old, this study remains one of the few real‐world, comparative evaluations of whole‐genome sequencing published, and is the only cancer‐focused study.^59^, ^60^, ^61^ Intervention patients included adults with locally advanced or metastatic, incurable disease, who initiated WGTA (*n* = 230).^15^, ^16^ Usual care controls were selected using matching and were eligible after receiving systemic therapy treatment for advanced stage disease (*n* = 13,279). WGTA occurred after developing an advanced cancer, often months or years after patients' initial cancer diagnosis, introducing the risk of immortal time bias. To minimize this risk, we indexed our analysis to biopsy date, which indicated the beginning of WGTA for intervention patients, rather than conducting comparative analysis from diagnosis date.^15^, ^16^ However, for usual care controls, biopsy date was unobserved and immortal time was unknown. We therefore applied two different methods to address immortal time bias: time‐distribution matching and MI. Following immortal time adjustment, we 1:1 matched treated patients and controls using genetic algorithm matching. A detailed description of our matching approach is published.^15^, ^16^ After matching, we conducted a cost‐consequence analysis. In this study, we focus on overall survival as the primary endpoint.

### 2.2 Data sources

We drew on de‐identified linked administrative datasets from BC Cancer, a population‐based provincial cancer registry that records demographic, disease, and mortality information for all cancer diagnoses in British Columbia, Canada. We identified intervention patients from the precision oncology program database. To assess systemic therapy and radiotherapy treatment histories, medical appointments, and tests received by the cohort, we obtained prescription records for drugs dispensed by BC Cancer pharmacies and drew on both the BC Cancer Radiotherapy database and Cancer Agency Information System (CAIS). Further details on all datasets used are published.^15^, ^16^

#### 2.2.1 Immortal time adjustment

**FIGURE 2:**
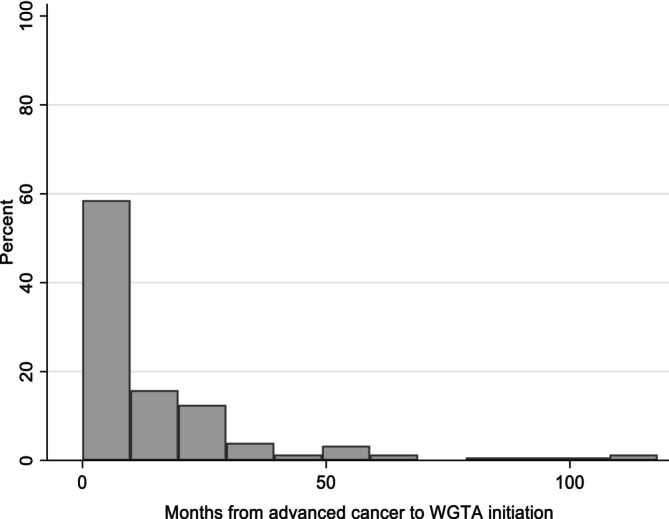
Distribution of length of time between date of advanced cancer and whole‐genome and transcriptome analysis (WGTA). The above figure reports a histogram showing months between WGTA initiation and treatment for patients who received an advanced systemic therapy treatment protocol. This figure was reproduced based on the following article with author permission: Weymann D, Laskin J, Jones SJ, Lim H, Renouf DJ, Roscoe R, et al. Matching methods in precision oncology: An introduction and illustrative example. Molecular genetics & genomic medicine. 2021;9 (1):E1554.

To adjust for immortal time, we first employed time‐distribution matching. We randomly assigned pseudo‐biopsy dates to population‐based controls based on the observed distribution of the length of time between advanced cancer date and biopsy date among intervention patients (reproduced in Figure 2). Following index date assignment, we excluded control patients who were ineligible for their assigned index date owing to death or censoring (*n* = 8055, 61%). We reestimated baseline covariates, conducted 1:1 matching, and performed outcomes analysis at this new index date. In sensitivity analysis, we then applied MI.

**FIGURE 3:**
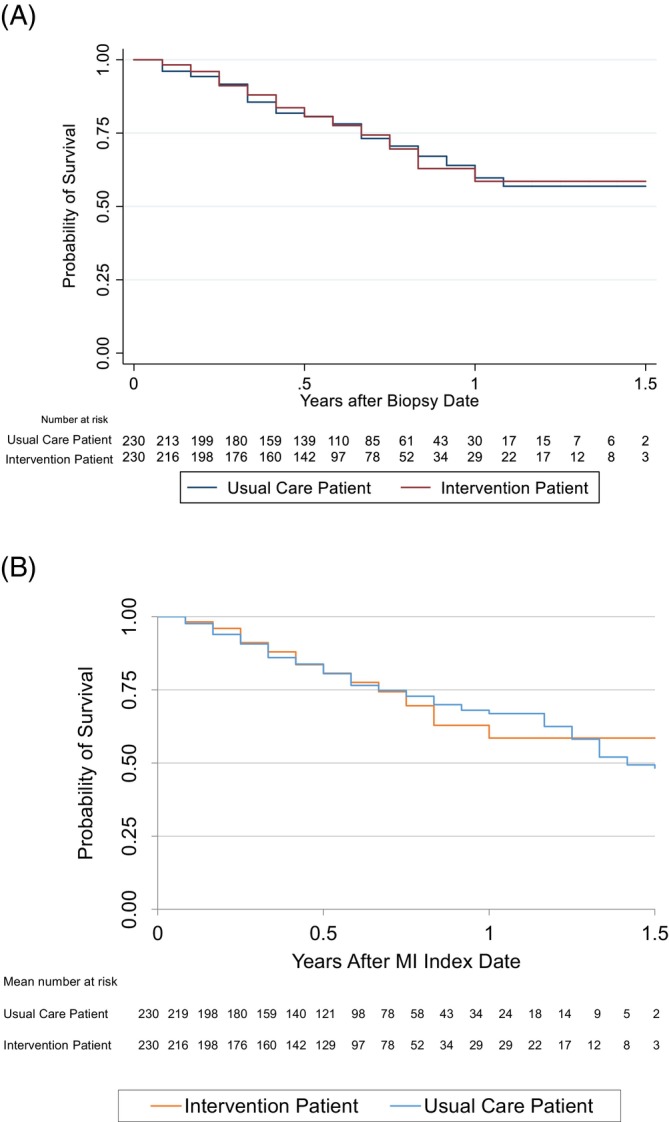
(A) Kaplan–Meier survival estimates for intervention patients and matched controls using time distribution matching to adjust for immortal time. (B) Kaplan–Meier survival estimates for intervention patients and matched controls using multiple imputation to adjust for immortal time. The above graphs depict patients' estimated probability of survival over time. Risk tables present the number of uncensored patients at risk of death at the beginning of each interval across groups. These figures were reproduced based on the following article with author permission: Weymann D, Laskin J, Jones SJ, Roscoe R, Lim HJ, Renouf DJ, et al. Early‐stage economic analysis of research‐based comprehensive genomic sequencing for advanced cancer care. Journal of community genetics. 2022;13 (5):523–38.

For MI, we considered missingness in the length of the immortal time period from advanced cancer date to biopsy date.^15^ Given these were missing by design with observed treatment status determining missingness, a MAR assumption was plausible.^46^ Among intervention patients, the length of the immortal time variable was continuous and right‐skewed (Figure 3). We therefore selected predictive mean matching as the univariate imputation method. Predictive mean matching is robust to deviations from the correct imputation model and non‐normality, and uses a two‐step process to impute missing values.^62^ First, a linear regression model is fit to obtain linear predictions. These predictions are then used as a distance measure to identify nearest neighbors from which to sample a complete value for a missing observation.

Predictive mean matching requires an additional decision beyond the generalized MI considerations already discussed, namely specifying the number of nearest neighbors. Based on past simulations showing that a larger number of nearest neighbors improves coverage and efficiency of effect estimates, we specified 10 nearest neighbors for imputation.^62^ Our imputation model considered all covariates factoring into subsequent matching and outcomes analysis and measurable at advanced cancer date, including: sex, age, rurality of geographic location, primary cancer type, year of initial cancer diagnosis, stage, tumor grade, and performance status at initial cancer diagnosis, and the length of time from initial cancer diagnosis to advanced cancer date. Survival did not factor into the imputation model given that inclusion of outcomes during imputation risks biasing final matched cohort analyses.^41^ We generated *n* = 10 MI datasets to reduce power‐fall off in alignment with past simulations.^54^ After imputation, we 1:1 matched patients and conducted survival analysis within each MI dataset at the new index date, date of advanced cancer adjusted for imputed immortal time. We combined results across MI datasets using Rubin's rules, applying a complementary log‐log transformation for normalizing survival probabilities and a logarithmic transformation for hazard ratios, and reported median p‐values for log‐rank tests.^55^, ^63^, ^64^

### 2.3 Survival analysis

After adjusting for immortal time and identifying matched cohorts, we estimated overall survival from index date until end of follow‐up, December 2015. We estimated Kaplan–Meier (KM) survival functions and used log rank tests to assess differences.^65^ To determine the hazard ratio (HR) associated with WGTA initiation compared to usual care, we estimated unadjusted Weibull regression models of probability of mortality. All survival analyses accounted for both censoring and weights related to matching with ties and replacement. We identified statistical significance using a threshold of *p* < 0.05. All analyses were conducted in R and Stata 15.^66^, ^67^

### 2.4 Bootstrap simulation study

To explore sensitivity of immortal time imputation to data missingness rates for varying sample sizes, we conducted a simulation study using nonparametric bootstrapping. Holding the number of complete cases fixed at either 230, as observed, or a randomly selected subset of 200, 150, 100, or 50 patients, we generated *n* = 10 MI datasets following the procedures detailed above. Within each dataset, we then used bootstrap simulation with replacement to vary the proportion of missing data from 98.3%, as observed, to 90%, 80%, 60%, or 50%. We generated 1000 replications for each. To assess performance, we measured changes in imputation results across scenarios by examining the mean imputed length of immortal time, $\bar{x}$, and the associated within‐ and between‐imputation variance, U and B. We estimated: (1) the mean imputed immortal time as x¯=∑m=110x¯m10, where ${\bar{x}}_{m}$ was the bootstrapped mean within each MI dataset; (2) the within‐imputation variance as $U=\frac{1}{m}\sum_{m=1}^{10}U_{m}$, where $U_{m}$ was the bootstrapped variance for ${\bar{x}}_{m}$ within each imputed dataset; and (3) the between‐imputation variance as $B=\frac{1}{m-1}\sum_{m=1}^{10}{\left({\bar{x}}_{m}-\bar{x}\right)}^{2}$, in alignment with Rubin's rules.

## 3 RESULTS

Our published evaluation identified 230 intervention patients and 13,279 control patients treated for advanced stage disease who were eligible for matching.^15^, ^16^ Following both time distribution matching and MI, all intervention patients were 1:1 genetic algorithm matched to 230 controls. Overall survival estimates after time distribution matching are reproduced in Figure 2A. We found that following immortal time adjustment, overall survival did not significantly differ across intervention patients and matched controls (*p* = 0.966). WGTA initiation irrespective of treatment change was not associated with a significant change in hazard of death compared to usual care (HR: 0.97, 95% CI: 0.68, 1.39).

Overall survival estimates after using MI to adjust for immortal time are reproduced in Figure 2B. Pooling across all imputed datasets, we once again detected no statistically significant overall survival differences across intervention patients and matched controls (*p* = 0.739) or changes in patients' hazard of death (HR: 1.04, 95% CI: 0.63, 1.69). While estimated overall survival probabilities and statistical hypothesis test results did not substantively change compared to time distribution matching, standard errors were higher for all point estimates. These findings suggest that MI may better represent uncertainty when adjusting for immortal time, by reflecting both variability across the observed cohort and uncertainty in the true value of immortal time.

### 3.1 Simulation results

**FIGURE 4:**
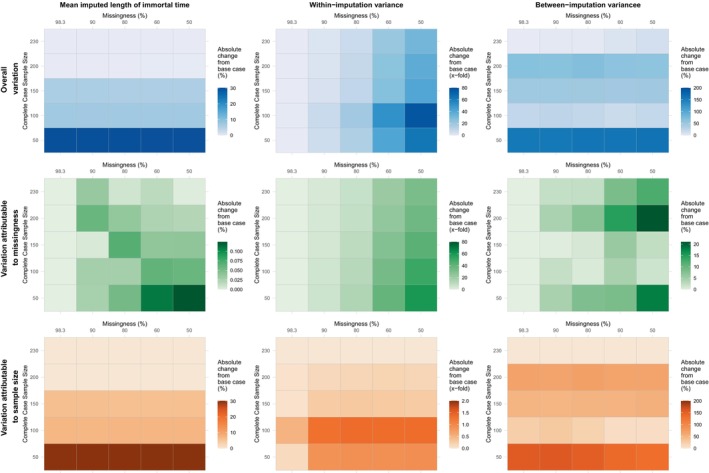
Gradient of change in mean imputed immortal time, within‐imputation variance, and between‐imputation variance for varying complete case sample sizes and missing data proportions. The above figures depict the observed gradient of change in each performance metric examined in simulation when varying either the sample size of complete cases upon which the imputation model is based, the percentage of missing data, or both simultaneously. Accurate interpretation of these results is dependent on the figure legend for each sub‐plot. Changes in mean imputed immortal time and between‐imputation variance are expressed as percentage changes and changes in within‐imputation variance are expressed as fold‐changes.

The results of our simulation study across each of the three metrics are in Figure 4. With 230 complete cases and a data missingness rate of 98.3%, the mean imputed immortal time was 20.27 days across MI datasets. The associated within‐variance estimate was 0.08, and the between‐variance estimate was 0.30. Mean imputed immortal time was robust to varying the proportion of missing data. Across simulations, the estimated mean changed by a maximum of 0.04% when the proportion of missing data varied and complete cases were fixed at 230. Mean imputed time was sensitive to the number of complete cases within the imputation model. The results were stable for as few as 100 complete cases, changing by a maximum of 7.23%. In our more finite samples of 50 complete cases, mean imputed times changed by up to 30.22%, resulting in a mean of 14.14 days.

Holding the number of complete cases fixed, as the proportion of missing data declined so did the number of available incomplete cases. Within‐imputation variance, which reflects sampling variance, showed greater sensitivity to sample size reductions for incomplete cases than between‐imputation variance. For 230 complete cases, as the proportion of missing data and available incomplete cases declined, within‐imputation variance increased from 0.08 to up to 2.70, and between‐imputation variance increased from 0.30 to 0.34. Both showed sensitivity to the number of complete cases informing imputation model estimates, with effects most pronounced for between‐imputation variance (Figure 4).

## 4 DISCUSSION

Failing to adjust for immortal time in comparative precision medicine evaluations can overestimate time‐dependent outcomes. Inaccurate estimation of these outcomes will lead to inappropriate causal inference. Landmark analysis, time‐distribution matching, time‐dependent analysis, and MI can each support mitigation of immortal time bias. Knowledge of corresponding strengths and limitations is critical to identifying the appropriate adjustment method. Landmark analysis is straightforward to apply and by far the most common approach in cancer‐related evaluations, but results and conclusions are sensitive to the choice of landmark time and information loss across both intervention and control patients can drive estimation bias.^17^, ^25^ Time‐distribution matching reduces information loss and is especially relevant for comparisons of groups who did not experience the same index date.^27^, ^28^, ^29^, ^30^ Yet this approach involves non‐informative date assignment that can also bias results.^19^ Time‐dependent analysis accounting for time‐varying treatment status is a common alternative that performs well in previous simulation and real‐world validation studies.^19^, ^31^, ^68^ Reliability requires consideration and adjustment for delayed entry bias and time‐varying confounding, particularly for evaluations of progressive diseases.

Our proposed application of MI offers theoretical advantages to existing methods. MI avoids false precision and improves estimation of uncertainty, through measuring uncertainty in the true length of immortal time in addition to uncertainty across the observed cohort. Index dates imputed through MI explicitly consider intervention and control patient characteristics and their association with immortal time, without inadvertently introducing delayed entry bias. In our precision medicine case study, estimated variability measures increased for MI compared to time distribution matching. In simulation, mean imputed immortal times and associated between‐imputation variance were stable for varying rates of missing data, consistent with past literature recommending that the proportion of missing data not be used to guide MI decisions.^69^ Between‐imputation variance was sensitive to changes in the number of complete cases, whereas within‐imputation variance was sensitive to the sample size of incomplete cases. Our findings align with theoretical expectations that MI will allow for increased uncertainty through estimating multiple values for a missing value.^37^ Although the magnitude and statistical significance of effect estimates did not substantively vary in our real‐world example, it is possible that other precision medicine evaluations will be more sensitive to increased uncertainty. In these settings, higher uncertainty may affect statistical significance of findings and change policy recommendations for decision‐makers. Additional real‐world applications of our proposed MI approach would be beneficial to enhance generalizability.

### 4.1 Limitations

Our study is subject to limitations. Our overview of existing analytic solutions for addressing immortal time bias is non‐exhaustive and instead focuses on the most commonly applied adjustment methods to date. Several established alternatives exist that may outperform these methods, such as the recently developed cloning technique which uses the framework of emulated trials to address immortal time bias, or the prevalent new‐user cohort design proposed for single‐arm trial evaluations.^14^, ^70^, ^71^ The latter relies on either time‐based or prescription‐based exposure definitions to determine the point of comparability for intervention and control patients, and draws on longitudinal matching methods to address time‐varying confounding. Establishing the relative performance of these competing approaches versus MI is an important avenue for further investigation. Future research may also consider the applicability and performance of deep learning alternatives to MI for imputing immortal time, such as denoising autoencoders.^72^ Our case study of MI focuses on a real‐world application in which the true treatment effect is unknown and uncertainty remains regarding whether immortal time bias was fully eliminated. While this applied study establishes the feasibility of using MI for imputing immortal time, improves our understanding of real‐world performance compared to time‐distribution matching, and uses bootstrap simulation to examine the influence of sample sizes and missing data rates on MI results, a future more expansive simulation study is warranted. Simulation can enable characterization of relative performance for MI versus other immortal time adjustment methods subject to different data generation assumptions and imputation model specifications.

## 5 CONCLUSION

Immortal time challenges the validity of comparative evidence for precision medicine decision‐making. The prevalence of single‐arm, master protocol trials for evaluating targeted therapies necessitates real‐world observational studies at increased risk of immortal time bias. Delays in accessing genomic testing and subsequent targeted treatment may further augment this risk. Regardless of the method of adjustment, precision medicine evaluations require explicit consideration of time‐related biases. This study provides guidance for mitigating immortal time bias in precision medicine to support reliable regulatory and resource allocation decisions.

## AUTHOR CONTRIBUTIONS

Study conception and design: All authors. Statistical analysis: All authors. Drafting of the manuscript and final approval: All authors. Guarantor of work: All authors.

## CONFLICT OF INTEREST STATEMENT

Dean A. Regier has received travel funding from Illumina; his institution has received research funding for a project from Roche Canada. Deirdre Weymann co‐directs IMPRINT Research Consulting; has consulted for Roche Canada and AstraZeneca Canada; and has received travel funding from Illumina.

## Supporting information


**Data S1.** Supporting Information.

## Data Availability

Data sharing is not applicable to this article as no datasets were newly generated or newly analyzed during the current study.
