# Supplementary material for: Addressing immortal time bias in precision medicine: Practical guidance and methods development
Source: Health Serv Res. 2024 Sep 3;60(1):e14376. doi: 10.1111/1475-6773.14376 (PMC11782076; doi:10.1111/1475-6773.14376)
Supplement: Supplementary file 1 — Data S1. Supporting Information. [file HESR-60-0-s001.docx]

Addressing immortal time bias in precision medicine: Methods development and practical guidance

**SUPPLEMENTAL MATERIALS**

**Rubin’s rules for combining estimates across multiply imputed datasets:**

Apply normalizing transformation, if necessary:

(1) $\hat{G(x)}=G(\hat{f\left( x \right)})\sim N(\mu,\sigma)$

Let the i^th^ estimate of $\beta$be $\hat{\beta_{i}}$ such that the mean estimate is:

(2) $\bar{\beta}_{MI}=\frac{\sum_{i=1}^{N} \beta_{i}}{N}$

Calculate the variance of each i^th^ estimate:

(3) $Var_{within}= \frac{\sum_{i=1}^{N} SE_{i}^{2}}{N}$

Calculate the variance between imputations:

(4) $Var_{between}= \frac{\sum_{i=1}^{N} \left( \beta_{i}-\bar{\beta}_{MI} \right)^{2}}{N-1}$

Combine the estimates:

(5) $Var_{MI}=Var_{within}+\left( 1+\frac{1}{N} \right)Var_{between}$

Back-transformation, if necessary.
